# Deficiency of glucocorticoid receptor in bone marrow adipocytes has mild effects on bone and hematopoiesis but does not influence expansion of marrow adiposity with caloric restriction

**DOI:** 10.3389/fendo.2024.1397081

**Published:** 2024-06-03

**Authors:** Rebecca L. Schill, Jack Visser, Mariah L. Ashby, Ziru Li, Kenneth T. Lewis, Antonio Morales-Hernandez, Keegan S. Hoose, Jessica N. Maung, Romina M. Uranga, Hadla Hariri, Isabel D. K. Hermsmeyer, Hiroyuki Mori, Ormond A. MacDougald

**Affiliations:** ^1^ Department of Molecular & Integrative Physiology, University of Michigan, Ann Arbor, MI, United States; ^2^ Department of Periodontics and Oral Medicine, University of Michigan School of Dentistry, Ann Arbor, MI, United States; ^3^ Department of Internal Medicine, University of Michigan, Ann Arbor, MI, United States

**Keywords:** glucocorticoids (GC), glucocorticoid receptor (GR), bone marrow adipose tissue (BMAT), caloric restriction (CR), bone, hematopoiesis

## Abstract

**Introduction:**

Unlike white adipose tissue depots, bone marrow adipose tissue (BMAT) expands during caloric restriction (CR). Although mechanisms for BMAT expansion remain unclear, prior research suggested an intermediary role for increased circulating glucocorticoids.

**Methods:**

In this study, we utilized a recently described mouse model (*BMAd-Cre*) to exclusively target bone marrow adipocytes (BMAds) for elimination of the glucocorticoid receptor (GR) (i.e. *Nr3c1*) whilst maintaining GR expression in other adipose depots.

**Results:**

Mice lacking GR in BMAds (*BMAd-Nr3c1* ^-/-^) and control mice (*BMAd-Nr3c1* ^+/+^) were fed *ad libitum* or placed on a 30% CR diet for six weeks. On a normal chow diet, tibiae of female *BMAd-Nr3c1^-/-^* mice had slightly elevated proximal trabecular metaphyseal bone volume fraction and thickness. Both control and *BMAd-Nr3c1^-/-^* mice had increased circulating glucocorticoids and elevated numbers of BMAds in the proximal tibia following CR. However, no significant differences in trabecular and cortical bone were observed, and quantification with osmium tetroxide and μCT revealed no difference in BMAT accumulation between control or *BMAd-Nr3c1* ^-/-^ mice. Differences in BMAd size were not observed between *BMAd-Nr3c1^-/-^* and control mice. Interestingly, *BMAd-Nr3c1^-/-^* mice had decreased circulating white blood cell counts 4 h into the light cycle.

**Discussion:**

In conclusion, our data suggest that eliminating GR from BMAd has minor effects on bone and hematopoiesis, and does not impair BMAT accumulation during CR.

## Introduction

Adipocytes are widely distributed throughout the human body, and their physiological functions differ depending on location. Bone marrow adipose tissue (BMAT) is a unique adipocyte depot located within the medullary cavity of bones. While the presence of BMAT has been known since the late 19^th^ century, the physiological importance of BMAT remains incompletely understood. Humans are born with very little BMAT, but it gradually expands with age, and by adulthood, BMAT constitutes about 50-70% of total marrow volume (1). Two types of BMAT have previously been described (2). Constitutive BMAT (cBMAT) is located within the distal region of long bones and in caudal vertebrae, and as suggested by the name, remains constitutively present despite a wide variety of physiological interventions. Alternatively, regulated BMAT (rBMAT) is in proximal tibia and distal femur. rBMAT is typically seen as single cells or in small clusters of adipocytes interspersed with hematopoietic cellularity. rBMAT volume changes under a variety of physiological and pathological conditions. For example, cold exposure, acute myeloid leukemia, exercise, and lactation lead to decreased rBMAT volume (3). Alternatively, expansion of rBMAT is observed with type 1 and type 2 diabetes, obesity, growth hormone deficiencies, impaired hematopoiesis, osteoporosis, as well as caloric restriction (CR) (3). BMAT also functions to influence bone and hematopoiesis by serving as an endocrine organ, contributing to circulating concentrations of adiponectin, stem-cell factor, leptin, and receptor activator for NF-κB (RANK) ligand (4–6).

Bone is a dynamic organ that responds to local and systemic stimuli to regulate resorption and formation of bone. Most human and mouse data suggest an inverse relationship between bone marrow adiposity and bone mineral density (7); however, this is not always the case. In C57Bl/6J mice, long-term high fat diet leads to increased BMAT with variable loss to trabecular and cortical bone mass (8, 9). Following bariatric surgery in mice, there is a dramatic loss of BMAT, in addition to a loss trabecular and cortical bone mass (10). Recent work demonstrates that BMAT has a negative effect on bone mass, although, under conditions of energy deficit, BMAT lipolysis helps to maintain bone mass (11). BMAds arise from skeletal stem cells, which also give rise to osteoblasts. Fate determination is controlled by several key transcription factors including runt-related transcription factor 2 (Runx2) and osterix (Osx), which promote osteoblastogenesis (12, 13) and proliferator-activated receptor gamma (PPARγ) and CCAAT/enhancer-binding protein alpha (C/EBPα), which promote adipogenesis (14). The bone marrow microenvironment is complex. In addition to impacting bone health, BMAds have also been shown to influence hematopoiesis. Several studies have suggested that BMAds are a negative regulator of hematopoiesis (15, 16). However, other studies suggest that BMAds support the function of hematopoietic cells (11, 17, 18). Thus, interactions between BMAT and other cell types within the marrow are complex and remain poorly understood.

CR has been shown to increase lifespan and improve overall metabolic health (19). Unlike white adipose tissue (WAT), BMAT paradoxically increases during CR (20), suggesting that BMAT and WAT are metabolically and functionally distinct. The physiological purpose for increased BMAT with CR is unknown; however, a recent study from *Li et al.* demonstrates that lipolysis of BMAds helps to fuel the bone and myelopoiesis during times of CR (11). M olecular signals that lead to these changes in BMAT volume remain poorly understood. Previous hypotheses have included potential roles for leptin, estradiol, fibroblast growth factor 21, ghrelin, and cortisol (21–25). Previously, *Cawthorn et al.* (26) showed that CR in female mice leads to BMAT increase, without changes in circulating leptin concentrations. Using mice and rabbits, these studies also showed that BMAT expansion was observed only when circulating corticosterone concentrations were elevated, implying a potential role for glucocorticoids in BMAT expansion following CR.

Glucocorticoids (GC) are corticosteroids that are essential for vertebrate biology. Due to their anti-inflammatory effects, they are widely used to treat a variety of inflammatory diseases. Glucocorticoids are mainly synthesized by the cortex of the adrenal gland. Production of GCs is regulated by the hypothalamic-pituitary-adrenal axis. Mechanisms of action by GCs are also tightly regulated by enzymatic conversion between active and inactive forms. Two enzymes regulate the conversion between active and inactive GCs. 11β-hydroxysteroid dehydrogenase 1 (11β-HSD1) catalyzes the conversion of inactive cortisone (11-dehydrocorticosterone in mice) to active cortisol (corticosterone in mice), while 11β-HSD2 performs the opposite conversion (27, 28). GCs bind to the glucocorticoid receptor (GR, gene name *Nr3c1)*, a member of the nuclear receptor family. This receptor functions to regulate GC-responsive genes. Furthermore, activation of GRs is regulated by its subcellular distribution. When unbound, GR resides in the cytosol as a monomer, stabilized by several heat shock proteins. Once bound by a GC, a conformation change occurs, leading to exposure of two nuclear localization signals, at which point GR is transported to the nucleus. Once nuclear, GR directly binds DNA through GC-response elements. The effects of GC on adipose tissue are complex. Under most circumstances, GC stimulates fatty acid uptake and lipolysis in adipocytes (29, 30). Chronic GC treatment leads to metabolic impacts such as insulin resistance, dyslipidemia, and obesity (31). GC are present in almost all adipocyte differentiation protocols (32). In particular, the use of dexamethasone, a synthetic GR ligand, is common. Several groups have used adipocyte GR-knockout models with mixed results (31). Most studies demonstrate that GR is not required for development or maintenance of adipose depots (33–35). However, some studies suggest it may play an important role during high-fat diet feeding (36). Importantly, none of these studies have investigated the impact of adipocyte GR on bone biology.

To investigate the role of GC in BMAT expansion, we utilized a previously described BMAd-specific Cre mouse model to knock out GRs in BMAds (11). Female *BMAd-Nr3c1^-/-^* mice had a small but significant increase in trabecular bone volume fraction (Tb. BV/TV), trabecular thickness, and distal tibial bone volume (BV). Male *BMAd-Nr3c1^-/-^* mice did not show changes in bone parameters. Loss of GR in BMAds did not alter the response of young or adult male mice to CR. However, *BMAd-Nr3c1^-/-^* mice had decreased circulating white blood cells in the light cycle without changes to hematopoietic progenitor populations, suggesting that GRs in BMAds may play a regulatory role in hematopoiesis.

## Materials and methods

### Mouse generation, care, and housing

Generation and validation of the BMAd-specific Cre model were performed as previously described (11). By monitoring the conversion of cell membrane-localized tdTomato to cell membrane-localized EGFP, Cre efficiency was determined to be ~80% in both male and female mice over 16 weeks of age with one Cre allele, and over 90% with two Cre alleles (11). To generate *BMAd-Nr3c1* ^-/-^ mice, BMAd-specific Cre mice (11) were crossed to mice containing *loxP* sites flanking exon 3 of the *Nr3c1* gene (Jax Strain #: 021021. ID: B6. Cg-*Nr3c1^tm1.1Jda/J^* (37)). Control *BMAd-Nr3c1* ^+/+^ mice contain *Osterix-Flpo* and *Flp-activated adiponectin-CRE* but not the floxed *Nr3c1*. Since *BMAd-Cre* and *Nr3c1* ^-/-^ mice were initially obtained on mixed genetic backgrounds, and because a systematic breeding to congenicity was not performed, we monitored the background strain of *BMAd-Nr3c1* ^-/-^ (n=5; Transnetyx Inc, Cordova, TN). Our results demonstrated that these mice have an average observed frequency of the following sub-strains: 63.9% C57BL/6J, 13.2% C57BL/6NJ, and 33.4% C57BL/6. Three of the five mice demonstrated an observed frequency average of 17.6% 129S strain, with two mice showing nonsignificant amounts of the 129S strain. Together, these data indicate that mice used in these studies had a predominately C57BL/6 background. Control mice used in these studies are *BMAd-Cre* mice lacking *loxP* sites in the *Nr3c1* gene. The presence of *loxP* sites and confirmation of recombination in BMAds was determined by PCR (see Genotyping and PCR). Mice were housed in a 12 h light/dark cycle in the Unit of Laboratory Animal Medicine at the University of Michigan, with free access to water. Unless indicated, mice were fed a normal chow diet (NCD) *ad libitum* (LabDiet 5LOD PicoLab). All procedures were approved by the University of Michigan Committee on the Use and Care of Animals.

### Genotyping and PCR

The presence of flanking *loxP* sites on exon 3 of the mouse *Nr3c1* gene was determined using PCR and the following primers:

Forward primer (Fwd): ATGCCTGCTAGGCAAATGAT

Reverse primer #1 (R1): TTCCAGGGCTATAGGAAGCA

Recombination and removal of exon 3 of the mouse *Nr3c1* gene was determined using PCR and the following primers:

Forward primer (Fwd): ATGCCTGCTAGGCAAATGAT

Reverse primer #2 (R2): TTAAGACAGTCGTCTGGAATTCC

### Caloric restriction

After acclimation to single housing and the control diet (D17110202; Research Diets) for two weeks, food intake was determined by giving a defined amount of food and weighing the remainder daily for 2 weeks. Individual adult male mice (starting age of 28-35 weeks, body weights of 26.5 g to 35.2 g) consumed approximately 2.42 g of food per day. As such, we provided 1.70 g of the nutrient-matched CR diet (D19051601; Research Diets) to mice daily to ensure 30% CR. Female mice consumed approximately 2.27 g of food per day and were therefore provided 1.59 g of the CR diet. Mice on CR consumed all the food provided and food was provided at ~2 pm daily.

### Histology

Tissue histology was performed as previously described (10). Briefly, soft tissues were fixed in 10% formalin for 24 hours and embedded in paraffin for sectioning. Bones were fixed in formalin for 24 hours, decalcified in 14% EDTA for a minimum of 2 weeks, with fresh EDTA provided every 48 hours. Following decalcification, bones were fixed an additional 24 hours with 10% formalin. Bones were then embedded in paraffin and sectioned to 5 μm. After staining with hematoxylin and eosin (H&E), sectioned tissue was imaged on an Olympus BX52 microscope.

### μCT analysis

Tibial bone parameters were measured using μCT as previously described (11, 38). Briefly, the entire tibia was scanned using a μCT system (μCT100 Scanco Medical, Bassersdorf, Switzerland). The following parameters were used: voxel size 12 µm, 70 kVp, 114 µA, 0.5 mm AL filter, and integration time 500 ms. Mid-cortical bone: a total of 30 slices (360 µm) were analyzed. The starting slice number was determined using the following equation: *Starting slice = [(Tib/fib junction slice #) – (growth plate slice #)] x 0.7 + (growth plate slice #).* Trabecular bone: a total of 50 slices (600 µm) were analyzed, initiated 5 slices distal to the proximal tibial growth plate. Distal cortical bone: slices were analyzed starting at the junction of the tibia and fibula and continuing to the distal end of the cortical bone, where distal trabecular bone is first observed (~450 slices, ~5.4 mm). Schematic of tibial bone locations use for µCT is present within relevant figures.

### Osmium tetroxide staining and BMAd quantification

Mouse tibiae were fixed for 24 hours in formalin, then decalcified using 14% EDTA as previously described (10). Osmium tetroxide staining and μCT was performed as previously described (2, 38).

### Circulating corticosterone measurements

Immediately following euthanasia, blood was harvested via cardiac punction, allowed to clot on ice for 2 hours and the serum was collected and stored at -80°C. To measure circulating corticosterone concentrations, an ELISA was performed per the manufacturer’s recommended protocols (Cayman Chemical, 501320, Ann Arbor, MI).

### Complete blood count

Blood was harvested from the tail of *BMAd-Nr3c1^-/-^* and *BMAd-Nr3c1^+/+^* mice and a complete blood count was performed by the University of Michigan Unit for Laboratory Animal Medicine Pathology Core using a Heska Element HT5 Veterinary Hematology Analyzer (Loveland, CO). Blood draws were performed at zeitgeber time 5 (ZT5; 10 am) and ZT17 (10 pm).

### Bone marrow cellular quantification

Bone marrow was extracted from the femurs and tibiae of *BMAd-Nr3c1^-/-^* and *BMAd-Nr3c1^+/+^* mice. Bones were crushed and incubated in Red Blood Cell lysis buffer (Sigma-Aldrich St. Louis, MO) for five minutes on ice. Bone marrow compartments were visualized by flow cytometry after staining with the following antibodies: LT-HSC/ST-HSC/MPP2/MPP3/MPP4 [B220-PerCP (RA3-6B2), CD3-PerCP (145-2C11), CD4-PerCP (GK1.5), CD8-PerCP (53-6.7), CD19-PerCP (6D5), Gr-1-PerCP (RB6-8C5), Ter119-PerCP (TER-119), Sca-1-PerCP-Cy5.5 (E13-161.7), c-Kit-APC-780 (2B8), CD150-PE-Cy7 (TC15-12F12.2), CD48-Alexa Fluor 700 (HM48-1), and Flt3-PE (A2F10.1)]; CMP/GMP/MEP [B220-PerCP (RA3-6B2), CD3-PerCP (145-2C11), CD4-PerCP (GK1.5), CD8-PerCP (53-6.7), CD19-PerCP (6D5), Gr-1-PerCP (RB6-Ter119-PerCP (TER-119), Sca-1-PerCP-Cy5.5 (E13-161.7), c-Kit-APC-780 (2B8), FcR II/III-Alexa Fluor 700 (93), CD34-FITC (RAM34), and IL-7R-PE-Cy7 (A7R34)]. All antibodies were used at 1:200 dilution except for CD34-FITC, which was used at 1:50 dilution. Populations were identified according to the following gating strategy: LT-HSC [Lineage-Sca1+cKit+ (LSK)CD48-CD150+Flt3-]; ST-HSC (LSK, CD48-CD150-Flt3-); MPP2 (LSK, CD48+CD150+Flt3-); MPP3 (LSK, CD48+CD150-Flt3-); MPP4 (LSK, CD48+CD150-Flt3+); CMP (Lineage-Sca1-cKit+CD34+ FcR II/IIImed); GMP (Lineage-Sca1-cKit+CD34+ FcR II/IIIhigh); MEP (Lineage-Sca1-cKit+CD34- FcR II/III-); CLP (Lineage-Sca1medcKitmedIL-7R+). DAPI (Sigma-Aldrich, St. Louis, MO) was used for dead cell exclusion. Data collection was performed using a Northern Lights (Cytek) flow cytometer. Data analyses were performed with FlowJo version 10 (LLC, Ashland, OR).

### Statistics

Significant differences between groups were assessed using a two-sample *t*-test or ANOVA with post-tests as appropriate: one-way ANOVA with Tukey’s multiple comparisons test or two-way ANOVA with Sidak’s multiple comparisons test. All analyses were conducted using the GraphPad Prism version 9. All graphical presentations are mean ± SD. For statistical comparisons, a *P*-value of <0.05 was considered significant.

## Results

### Deletion of the GR in BMAds does not alter BMAT but slightly increases bone mass of proximal and distal tibia of female mice

**Figure 1:**
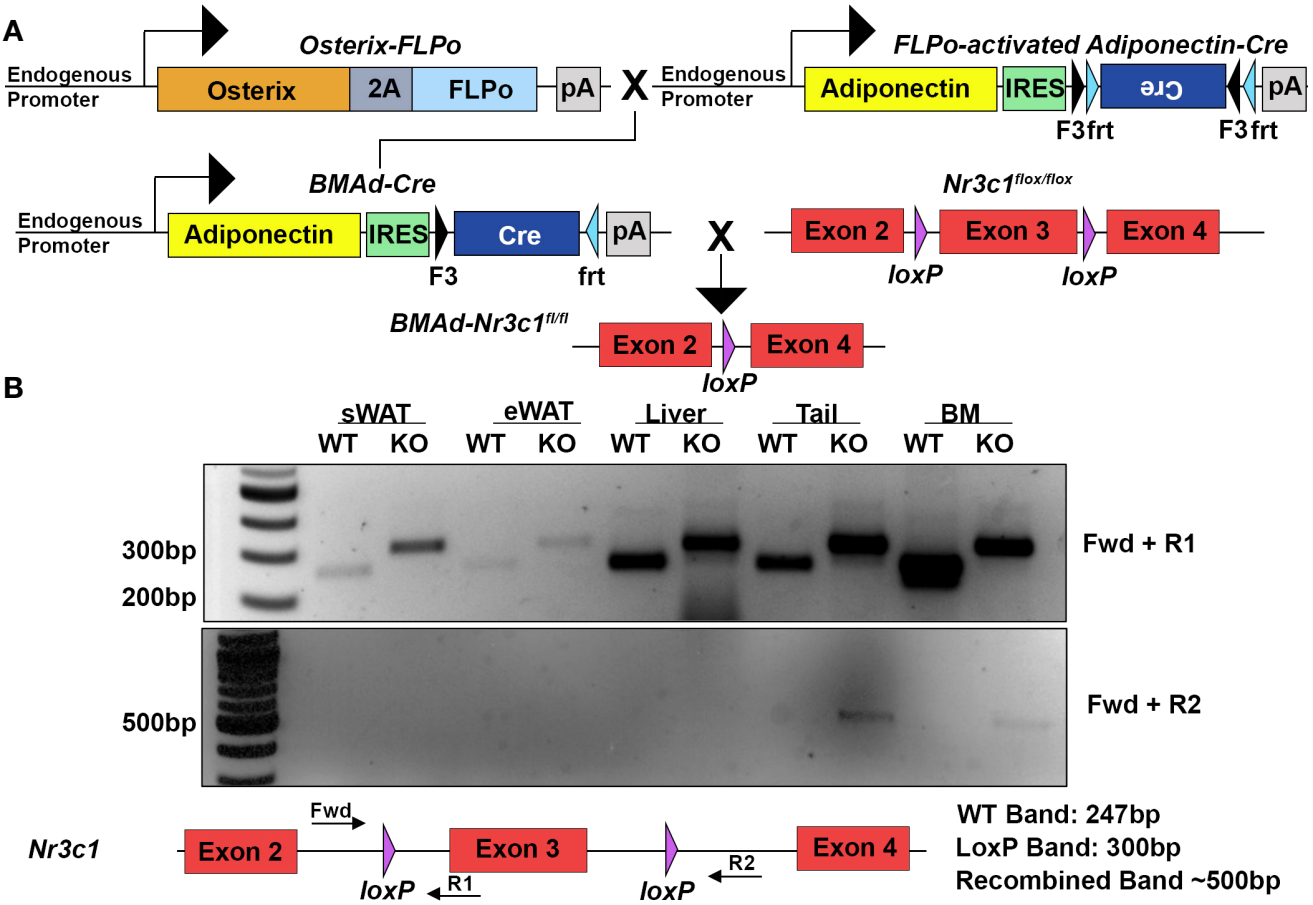
Deletion of GR specifically in BMAds using the BMAd-Cre mouse model. **(A)** Breeding strategy for generation of *BMAd-Nr3c1* ^-/-^ mice. **(B)** PCR amplification was used to detect the presence of loxP sites flanking exon 3 of *Nr3c1* (top panel, primers Fwd + R1). PCR products demonstrate presence of a band lacking exon 3 (bottom panel, primers Fwd + R2) in tail and bone marrow (BM) of *BMAd-Nr3c1* ^-/-^ (KO) mice but not in subcutaneous WAT (sWAT), epididymal (eWAT), or liver. Recombination was not detected in *BMAd-Nr3c1* ^+/+^ mice (WT).

To determine the roles of GR in BMAds we sought to selectively eliminate GR from BMAds while avoiding loss of GR in white adipocytes and osteoblasts. To do this, we utilized a recently described BMAd-specific Cre model (11). We crossed this *BMAd-Cre* line to mice containing *loxP* sites flanking exon 3 of the mouse GR gene (*Nr3c1*) (**Figure 1A**). We confirmed that recombination in *BMAd-Nr3c1^-/-^* mice occurred in caudal vertebrae and tibial bone marrow, where high amounts of BMAds are present (**Figure 1B**). No recombination was observed in subcutaneous WAT (sWAT), epididymal WAT (eWAT), or liver. In addition, RNA was isolated, converted to cDNA, and sequenced to confirm the presence of a product lacking exon 3 (not shown).

**Figure 2:**
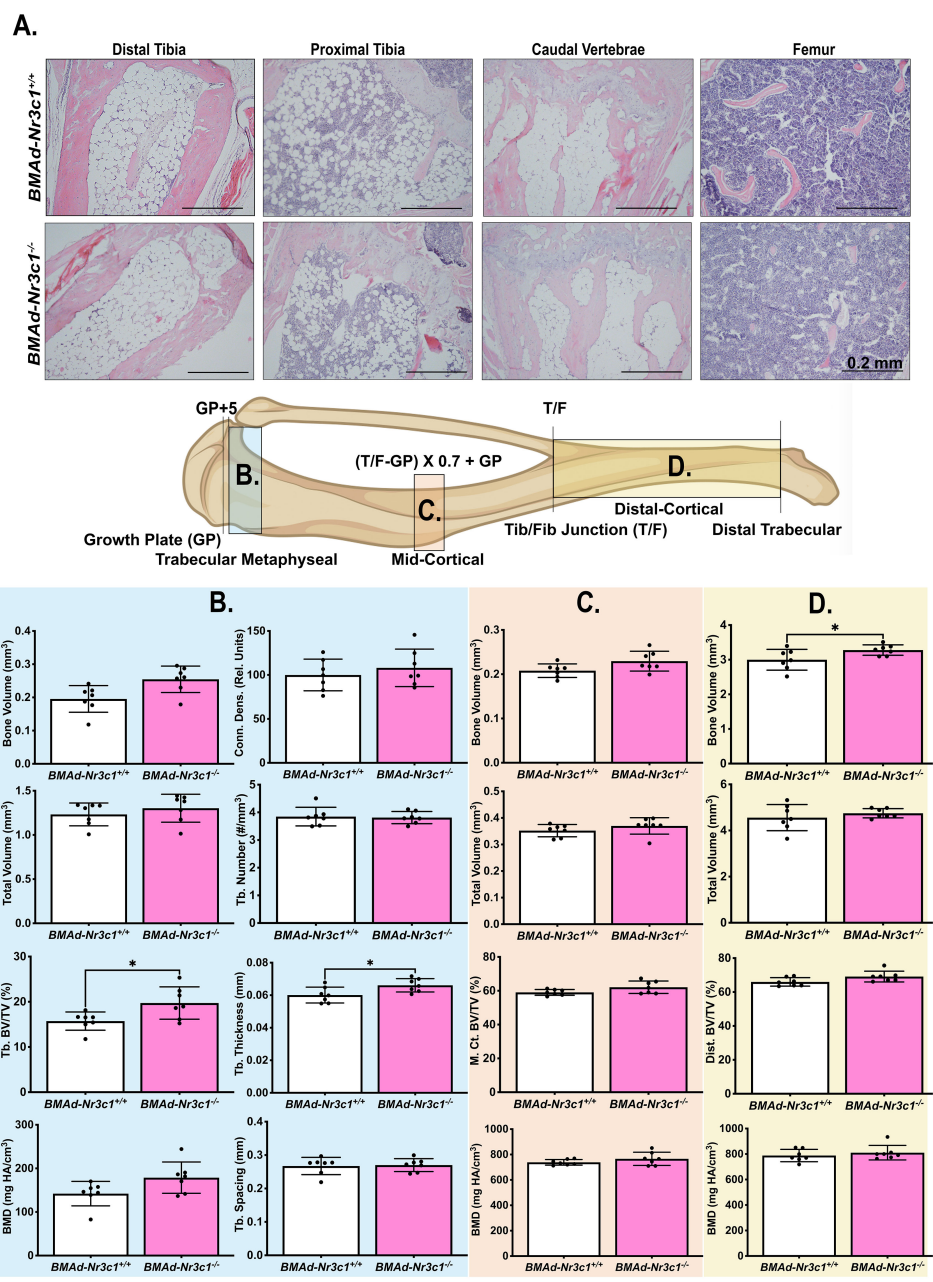
Female *BMAd-Nr3c1* ^-/-^ mice have slightly elevated tibial bone variables. **(A)** Female mice at 31–39 weeks of age were euthanized. Tibiae, tail, and femurs were decalcified, embedded, paraffin-sectioned, and stained with H&E. Representative photos are shown. Scale bar: 0.22 mm. **(B–D)** A schematic of the tibia illustrating locations of µCT slices. Tibiae were analyzed by µCT for **(B)** proximal trabecular metaphyseal, **(C)** mid-cortical, and **(D)** distal cortical bone variables. Tb: Trabecular, BV: Bone Volume, TV: Total Volume, Conn. Dens.: Connective Density, M. Ct.: Mid-Cortical, Dist., Ct.: Distal Cortical. Statistical analysis was performed using an unpaired t-test. *p<0.05.

We next investigated whether loss of GR in BMAds alters BMAT volume and/or tibial bone variables. Female *BMAd-Nr3c1^+/+^* and *BMAd-Nr3c1^-/-^* mice (31-39 weeks old) on a NCD were euthanized and a necropsy performed. As expected, no significant differences were observed in body weight (**Supplementary Figure 1A**) or tissue weights of the sWAT, periovarian WAT (poWAT), liver, or spleen of *BMAd-Nr3c1^-/-^* mice compared to controls (**Supplementary Figure 1B**). We also did not observe histological changes to the WAT (**Supplementary Figure 1C** **).** Next, we determined if loss of GR in BMAds alters BMAT volume. Histological analyses showed no observable differences in BMAT histology in the tibiae or caudal vertebrae of *BMAd-Nr3c1^-/-^* mice compared to controls (**Figure 2A**). Qualitative histological analysis suggested a slight reduction in the proximal trabecular metaphyseal bone in the femur of female *BMAd-Nr3c1^-/-^* mice. To determine quantitatively whether the lack of GR in BMAds alters bone parameters, we performed μCT on the tibiae of *BMAd-Nr3c1^+/+^* and *BMAd-Nr3c1^-/-^* mice. Female *BMAd-Nr3c1^-/-^* mice had a small but significant increase in proximal trabecular metaphyseal bone volume fraction and trabecular thickness (**Figure 2B**). No differences were observed in the mid-cortical region of the tibiae, where few BMAds are typically present (**Figure 2C**). Female *BMAd-Nr3c1^-/-^* mice also had a small but significant increase in cortical bone volume in the distal tibiae (**Figure 2D**). Although histological analysis suggested a slight reduction in trabecular bone in distal femur, male *BMAd-Nr3c1^-/-^* mice did not have differences in bone histology in tibiae or caudal vertebrae (**Supplementary Figure 2A**). Consistent with histological results, μCT did not reveal differences in b one parameters (**Supplementary Figures 2B-D**). These data indicate that at baseline, GR plays a dispensable role in size and number of BMAds and a minor role in inhibiting tibial bone mass in female but not male mice.

### Deletion of Nr3c1 in BMAds does not alter BMAT accumulation with CR

**Figure 3:**
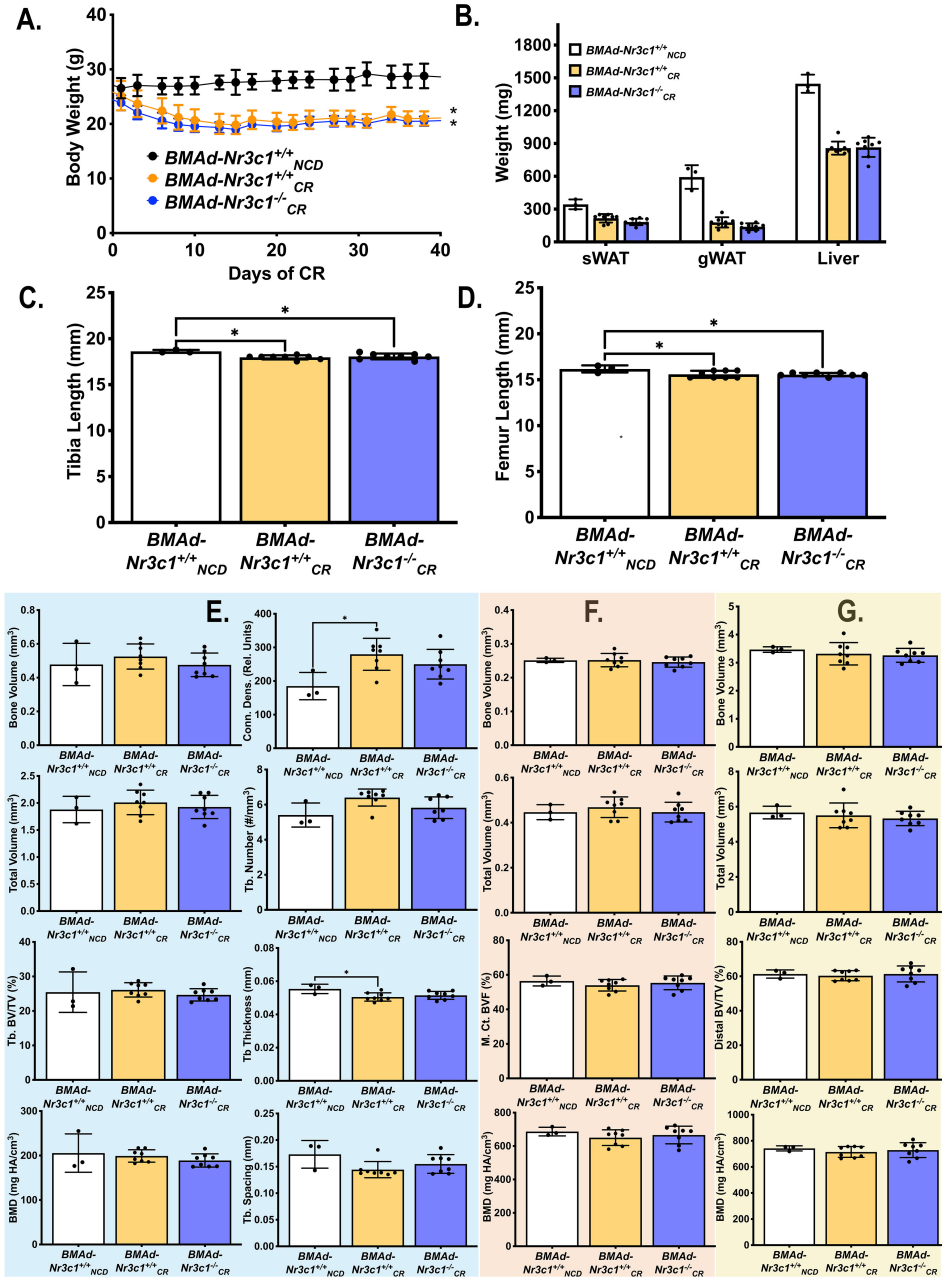
CR leads to decreased bone length in developing male mice independent of GR in BMAd. Male mice (10-16 weeks old) were fed *ad libitum* (NCD) or placed on a 30% CR diet for 6 weeks. **(A)** B ody weights of mice were measured throughout the experiment. Statistical analysis was performed using a two-way ANOVA. *p<0.05 compared to *BMAd-Nr3c1^+/+^_NCD_*. **(B)** At the time of euthanasia, tissue weights of sWAT, eWAT, and liver were measured. **(C, D)** Following necropsy, the length of the tibia **(C)** and femur **(D)** were measured. Tibiae were analyzed by μCT for **(E)** proximal trabecular metaphyseal, **(F)** mid-cortical, and **(G)** distal cortical bone variables. Tb: Trabecular, BV: Bone Volume, TV: Total Volume, Conn. Dens.: Connective Density, M. Ct.: Mid-Cortical, Dist. Ct.: Distal Cortical. Statistical analysis was performed using a one-way ANOVA with a Tukey multiple comparison *post hoc* test. *p<0.05.

In addition to increased BMAT volume, circulating concentrations of GC are elevated with CR in mice (26). We next sought to determine if GR is required for BMAT expansion following CR. Developing (10-16 weeks old) male *BMAd-Nr3c1^+/+^* and *BMAd-Nr3c1^-/-^* mice were fed *ad libitum* or were provided a 30% CR diet for 6 weeks. Because no significant changes in BMAT were observed in male *BMAd-Nr3c1^-/-^* mice at baseline (**Supplementary Figure 2**), all *BMAd-Nr3c1^-/-^* mice were placed on a 30% CR diet. As expected, CR mice had significant decreases in body and tissue weights (**Figures 3A, B**). CR mice also had significant decreases in length of tibiae and femurs (**Figures 3C, D**), with an increase in trabecular connective density (**Figure 3E**). Changes in the mid- or distal cortical bone were not observed (**Figures 3F, G**).

**Figure 4:**
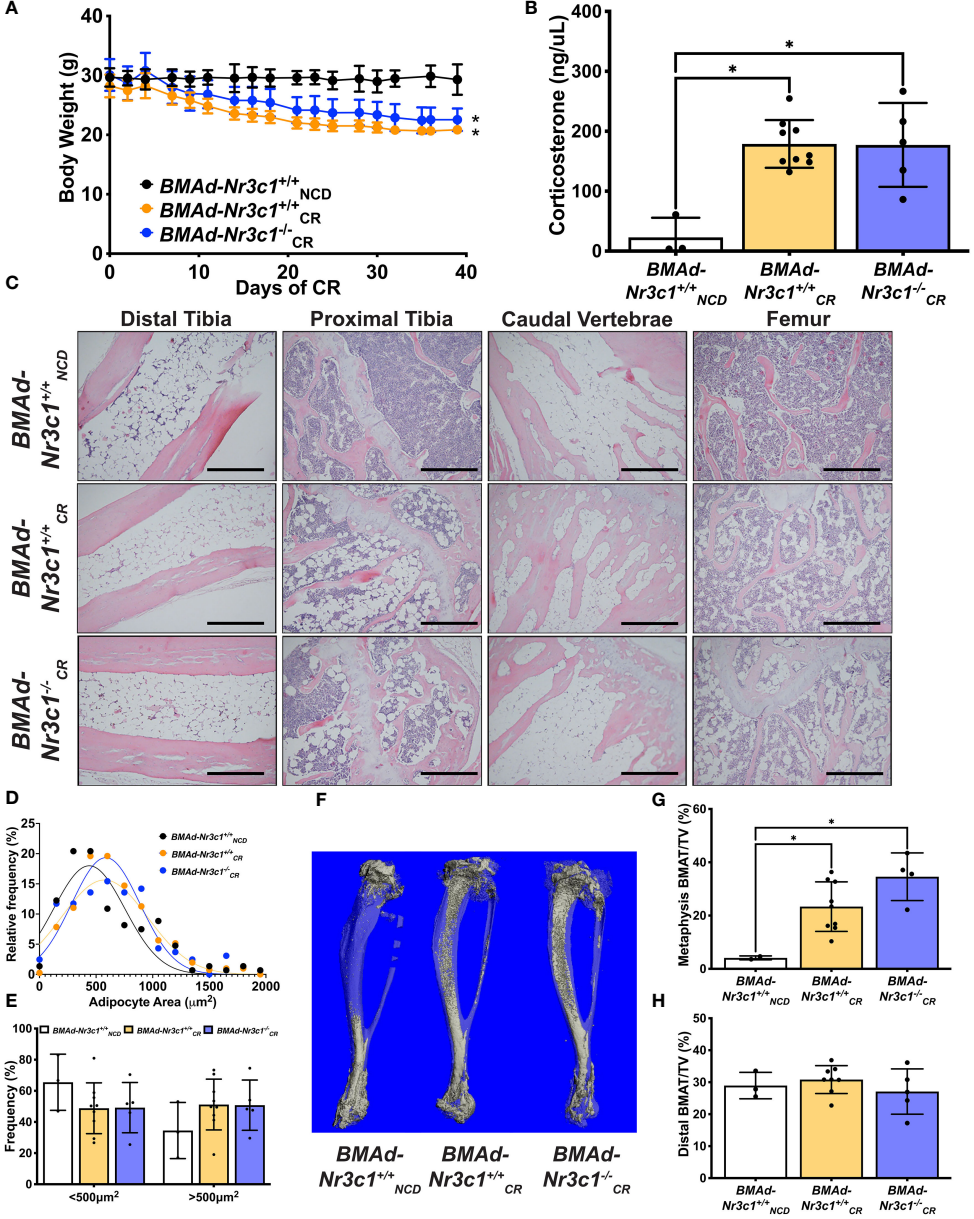
Loss of GR in BMAds of male mice does not alter BMAT responses to CR. Male mice (34-41 weeks old) were fed *ad libitum* or placed on a 30% CR diet for 6 weeks. **(A)** Body weight of mice was measured throughout the experiment. Statistical analysis was performed using a two-way ANOVA. *p<0.05 compared to *BMAd-Nr3c1^+/+^_NCD_*. **(B)** At the time of euthanasia, blood was isolated, and circulating corticosterone concentrations were measured. **(C)** Tibiae, caudal vertebrae, and femurs were decalcified, embedded, paraffin-sectioned, and stained with H&E. Representative photos are shown. Scale bar: 0.22 mm. **(D, E)** BMAd size was calculated using MetaMorph. **(F)** Using osmium tetroxide and μCT, a three-dimensional reconstruction of tibial BMAT was generated. BMAT volume in tibial **(G)** metaphasis and **(H)** distal cortical regions was determined.Statistical analysis was performed using a one-way ANOVA with a Tukey multiple comparison *post hoc* test. *p<0.05.

Due to decreased length of both the tibia and femur with CR, we concluded that CR blunts development of bone in growing mice. Therefore, we performed an additional CR study using adult male mice (34-41 weeks old) (**Figure 4**). CR mice lost ~26% of their body weight (**Figure 4A**) and had reductions in sWAT, eWAT, liver, and spleen mass (**Supplementary Figure 3A**). Importantly, we observed that CR induced a 7-fold increase in circulating corticosterone concentrations in both *BMAd-Nr3c1^+/+^* and *BMAd-Nr3c1^-/-^* adult mice (**Figure 4B**). Histological analysis showed CR leads to an increase in the number of BMAds in tibiae and femurs of mice placed on CR (**Figure 4C**), but differences in BMAT volume were not observed between *BMAd-Nr3c1^+/+^* and *BMAd-Nr3c1^-/-^* mice on CR. There were also no significant differences in adipocyte size in tibiae from *BMAd-Nr3c1^+/+^_CR_* and *BMAd-Nr3c1^-/-^_CR_* mice (**Figures 4D, E**). Quantification of BMAT volume using osmium tetroxide and μCT demonstrated a significant increase in proximal tibial BMAT with CR (**Figures 4F–H**). Neither CR nor the loss of GR in BMAds altered tibial bone parameters quantified by µCT (**Supplementary Figures 3B-D**).

### Deletion of Nr3c1 in BMAds alters circulating white blood cell counts

**Figure 5:**
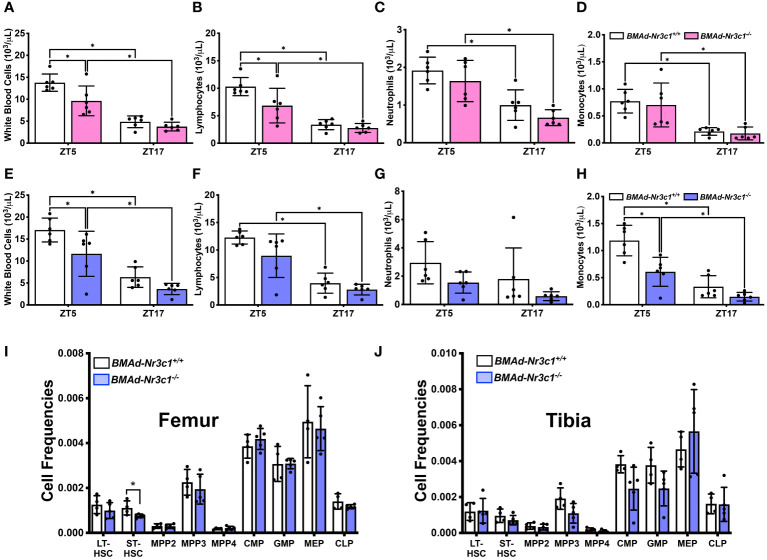
Loss of GR in BMAds reduces circulating white blood cell counts in female and male mice during the light cycle. Circulating blood cell populations were measured in **(A–D)** female and **(E–H)** male *BMAd-Nr3c1^-/-^* and control mice at ZT5 and, following a 48-hour recovery period, at ZT17. Statistical analyses of panels A-H were performed using a two-way ANOVA with a Sidak’s multiple comparison’s *post hoc* test. *p<0.05. Hematopoietic progenitor cell populations were measured in bone marrow isolated from the **(I)** femur and **(J)** tibia of *BMAd-Nr3c1^-/-^* or control mice. Statistical analysis of panels I and J was performed using an unpaired t-test. *p<0.05.

In addition to impacting bone biology, BMAds and GC have independent effects on hematopoiesis (39–42). To determine if loss of GR in BMAds impacts hematopoiesis, we measured complete blood counts from male and female *BMAd-Nr3c1^+/+^* and *BMAd-Nr3c1^-/-^* mice. Previous reports have shown that both GC and blood cell concentrations vary greatly with time of day (43, 44). Therefore, we measured blood cell parameters during the day (ZT5) and in the evening (ZT17). As expected, all blood cell populations were lower during the evening (**Figures 5A–H**). Our results demonstrated that both male and female *BMAd-Nr3c1^-/-^* mice have reduced white blood cell counts compared to *BMAd-Nr3c1^+/+^* mice (**Figures 5A, E**). Specifically, female *BMAd-Nr3c1^-/-^* mice showed a significant reduction in numbers of circulating lymphocytes (**Figure 5B**), whereas male mice showed a significant reduction in circulating monocytes (**Figure 5H**). Loss of GR in BMAds did not alter red blood cell parameters or platelets (**Supplementary Figure 4**). After observing changes to circulating blood cell populations, we next investigated whether this observation could be a result of changes to the bone marrow cell composition. Therefore, we measured hematopoietic progenitor cell populations within bone marrow compartments of femurs and tibiae. Our results demonstrated that *BMAd-Nr3c1^-/-^* mice had fewer short-term reconstituting hematopoietic stem cells (ST-HSC) in the femur. However, we did not observe changes in common myeloid progenitors (CMP) or common lymphoid progenitors (CLP). We also did not observe altered frequencies of progenitor cell populations in the tibia of *BMAd-Nr3c1^-/-^* mice compared to control (**Figures 5I, J**).

## Discussion

BMAds are a unique group of adipocytes residing within the bone marrow microenvironment. rBMAT volume expands under a variety of physiological and pathological conditions including CR (20) and anorexia nervosa (4, 45, 46). The molecular mechanisms during CR leading to increased BMAT are unknown. Previous studies using mouse models have shown that BMAT expansion occurs when circulating GC are elevated (26). Both excess synthetic and endogenous GCs are known to cause bone loss (47). In general, an inverse relationship exists between BMAT volume and bone mass, including in patients with anorexia nervosa (48–50). Together these results suggest that excess GC during CR may lead to increased BMAT and subsequent bone loss (51, 52). Indeed, work from *Pierce et al.* have shown that GR-deficiency in *Osx*-expressing cells led to loss of cortical and trabecular bone mass in mice fed *ad libitum* or CR (53). However, previous studies using adipocyte-specific deletion of GR have not investigated changes to bone mass (31, 33–35). Indeed, in these studies, the direct effects of GR in BMAd would not be distinguishable from potential indirect effects caused by the deletion of GR in other adipose depots.

In this study, our results suggest that GRs in BMAds are not required for the expansion of BMAT with CR. One explanation is that GR plays an important role in the early differentiation of BMAds, and the *BMAd-Cre* model used in these studies targets only mature BMAds (11). Another possibility is that the loss of GR in BMAds is compensated by other GC-responsive proteins such as the mineralocorticoid receptor (MR), which can also directly bind to GC. However, evidence for robust compensation by MR has not been observed in adipocyte-specific GR-KO models (33, 54). In the current studies, we also did not observe significant changes to MR expression levels in *BMAd-Nr3c1^-/-^* mice (data not shown). Several groups have targeted GC activity through knock-out of the enzymes involved in their activation, such as 11β-HSD1, a major regulator of the tissue-specific effects of GC (55, 56). Previous work has shown that global deletion of 11β-HSD1 produces a favorable metabolic state (57–59); however, the impact of 11β-HSD1-deficiency in BMAds has not been evaluated. With the recent invention of genetic tools like the *BMAd-Cre* mouse (11), future experiments can investigate this hypothesis that 11β-HSD1 is important for BMAT responses to GC. It is possible that GR in BMAds is important with other stressors that lead to BMAT expansion such as aging or obesity (60). Alternatively, it may be that elevated GC and BMAd-GR do not contribute to BMAT expansion following CR.

Whereas our results did not suggest a critical role for GR in BMAd in expansion of BMAT during CR, we did observe a small but significant increase in trabecular bone volume fraction and thickness as well as distal cortical bone volume in the tibiae of adult female *BMAd-Nr3c1^-/-^* mice at baseline. Sex-specific differences in bone parameters are well documented (61, 62), and 6 weeks of 30% CR did not lead to loss of tibial bone mass in adult male mice. Indeed, the clinical data surrounding bone loss from CR is variable. One study showed that six months of CR in young adults does not lead to significant bone loss (63). However, similar studies in adults show a significant reduction in bone following CR (64, 65). Amongst these conflicting findings, one consensus is that patients with anorexia nervosa are at an increased fracture risk due to bone loss (66–69). Of note, anorexia nervosa is a condition of more severe caloric deficiency than that achieved in most CR studies. In mouse and rabbit models, results have also been mixed. One common observation is that CR results in stunted bone growth and low bone mass in some regions of the skeleton of young mice (21) and rabbits (26). Indeed, our studies support this finding, which is why we then chose to investigate CR in adult mice (34+ weeks). In adult animals, most studies suggest that CR leads to bone loss (21, 70). However, results have been variable both in terms of the severity of bone loss and the location of bone loss within the skeleton (21, 70). One study showed that bone loss occurs with 6 weeks of CR, even when performed in combination with exercise (71), which typically positively supports bone health (72). Interestingly, some studies suggest that long-term CR may protect against or delay age-related bone loss (73, 74). Several factors could influence the impact of CR on bone mass including sex, age, length of CR, strain of mice, and region of the skeleton being investigated. While our CR diet includes micronutrient supplementation, in other dietary restriction studies (21), the availability of calcium and other essential minerals is of concern.

Due to its location within the marrow niche, as well as its endocrine functions, many studies have aimed to investigate the roles of BMAd on hematopoiesis. Several studies suggest an inverse relationship between BMAT and hematopoiesis (10, 15, 16, 18). However, others suggest a supportive role for BMAds in hematopoiesis (11, 18, 75). In our study, loss of GR from BMAds led to a significant decrease in circulating white blood cells. Female *BMAd-Nr3c1^-/-^* mice showed a decrease in lymphocytes, while male mice showed a significant reduction in monocytes. These changes were only significant when the blood draw was performed during the day (ZT5). Confirming previous studies showing that hematopoiesis is circadian (43), nearly all blood cell populations were reduced when the blood draw was performed at night (ZT17). We did not observe changes in circulating red blood cells in *BMAd-Nr3c1^-/-^* mice. While GR has previously been shown to mediate stress erythropoiesis (41), we hypothesize that the lack of changes in circulating red blood cells in *BMAd-Nr3c1^-/-^* mice is because this process mostly occurs in the spleen (76). In WAT, adipocyte GR suppresses the immune system, maintaining immune homeostasis (77, 78). While circulating white blood cells were decreased during the day in *BMAd-Nr3c1^-/-^* mice, we did not observe differences in the abundance of bone marrow progenitor cell populations in the tibiae of *BMAd-Nr3c1^-/-^* mice. We observed a subtle but significant change in ST-HSCs in the femur of *BMAd-Nr3c1^-/-^* mice, but the frequency of CMP or CLP populations was not different. BMAds locally interact with hematopoietic cells and contribute to whole-body metabolism through the secretion of adipokines (79, 80). Lipodystrophic A-ZIP/F1 mice, which lack BMAT, have delayed hematopoietic regeneration in the long bones following irradiation (5), a process that involves the secretion of stem cell factor (SCF) from BMAds (5, 80, 81). Loss of SCF from BMAT reduces the bone marrow cellularity, hematopoietic stem and progenitor cells, common myeloid progenitors, megakaryocyte-erythrocyte progenitors, and granulocyte-monocyte progenitors (81). Supporting this hypothesis, there is a depletion of BMAT accompanied by a decrease in bone marrow erythroid cells and anemia following bariatric surgery (10). Our data suggests that loss of GR from BMAd lowers circulating white blood cells without altering bone marrow hematopoietic progenitor cell populations.

Our experiments demonstrate that loss of GR in BMAd of female mice led to a small but significant increase in trabecular bone volume fraction and trabecular thickness. Loss of GR in BMAd of male mice showed no changes to bone parameters. CR in young mice resulted in a decrease in tibial and femur lengths as sufficient caloric intake is important for growth in developing bones. CR studies in both young and adult male mice demonstrated that GR in BMAd is not required for BMAT expansion following CR. This study has several limitations. One caveat to the *BMAd- Cre* mouse model is that the insertion of a flipped Cre gene in the 3’ untranslated region of adiponectin leads to a small, but significant, decrease in circulating adiponectin concentrations (11). To confirm that excess GC do not work directly on BMAds to promote BMAT expansion during CR, future mouse models should target other aspects of the GC pathway, such as deletion of MR or 11β-HSD1. It is also highly likely that GC induce BMAT expansion by promoting the differentiation of new adipocytes, and the *BMAd-Cre* model used herein targets mature adipocytes. In conclusion, our data suggest that BMAd-GR is not required for BMAT expansion following CR.

## Data availability statement

The raw data supporting the conclusions of this article will be made available by the authors, without undue reservation.

## Ethics statement

The animal study was approved by Institutional Animal Care & Use Committee University of Michigan. The study was conducted in accordance with the local legislation and institutional requirements.

## Author contributions

RS: Conceptualization, Data curation, Formal analysis, Funding acquisition, Investigation, Writing – original draft, Writing – review & editing. JV: Data curation, Writing – review & editing. MA: Data curation, Writing – review & editing. ZL: Data curation, Writing – review & editing. KL: Data curation, Writing – review & editing. AM-H: Data curation, Writing – review & editing. KH: Data curation, Writing – review & editing. JM: Data curation, Writing – review & editing. RU: Data curation, Writing – review & editing. HH: Data curation, Writing – review & editing. IH: Data curation, Writing – review & editing. HM: Data curation, Writing – review & editing. OM: Writing – original draft, Writing – review & editing.

## Supplementary material

Supplemental Figure 1 Deletion of GR from BMAds of female mice does not alter WAT, liver, or spleen weights. Female mice at 31–39 weeks of age were euthanized and **(A)** body weights and **(B)** tissue weights of sWAT, poWAT, liver, and spleen were determined. Statistical analysis was performed using an unpaired t-test. **(C)** sWAT and poWAT were fixed, paraffin-sectioned, and stained with H&E. Scale bar: 0.22 mm.

Supplemental Figure 2 Deletion of GR from BMAds of male mice does not alter BMAT or bone variables. **(A)** Male mice at 29–36 weeks of age were euthanized. Tibiae, caudal vertebrae, and femurs were decalcified, embedded, paraffin-sectioned, and stained with H&E. Representative photos are shown. Scale bar: 0.22 mm. Tibiae were analyzed by μCT for (B) proximal trabecular metaphyseal, **(C)** mid-cortical, and **(D)** distal cortical bone variables. Tb: Trabecular, BV: Bone Volume, TV: Total Volume, Conn. Dens.: Connective Density, M. Ct.: Mid-Cortical, Dist. Ct.: Distal Cortical. Statistical analysis was performed using an unpaired t-test.

Supplemental Figure 3 Calorie restriction of *BMAd-Nr3c1^-/-^* mice leads to loss of WAT but does not alter tibial bone variables. Male mice were euthanized and **(A)** tissue weights of sWAT, eWAT, liver, and spleen were determined. Statistical analysis was performed using a one-way ANOVA with a Tukey multiple comparison post hoc test. *p<0.05. Tibiae were analyzed by μCT for **(B)** proximal trabecular metaphyseal, **(C)** mid-cortical, and **(D)** distal cortical bone variables. Tb: Trabecular, BV: Bone Volume, TV: Total Volume, Conn. Dens.: Connective Density, M. Ct.: Mid-Cortical, Dist. Ct.: Distal Cortical.

Supplemental Figure 4 Deletion of GR from BMAds does not alter circulating red blood cell populations. Circulating blood cell populations were measured in **(A–D)** female and **(E–H)** male *BMAd-Nr3c1^-/-^* and control mice at ZT5 and, following a 48-hour recovery period, at ZT17. Statistical analysis was performed using a one-way ANOVA. *p<0.05.
