# Supplementary figures and images for: Deficiency of glucocorticoid receptor in bone marrow adipocytes has mild effects on bone and hematopoiesis but does not influence expansion of marrow adiposity with caloric restriction
Source: Front Endocrinol (Lausanne). 2024 Jun 3;15:1397081. doi: 10.3389/fendo.2024.1397081 (PMC11180776; doi:10.3389/fendo.2024.1397081)

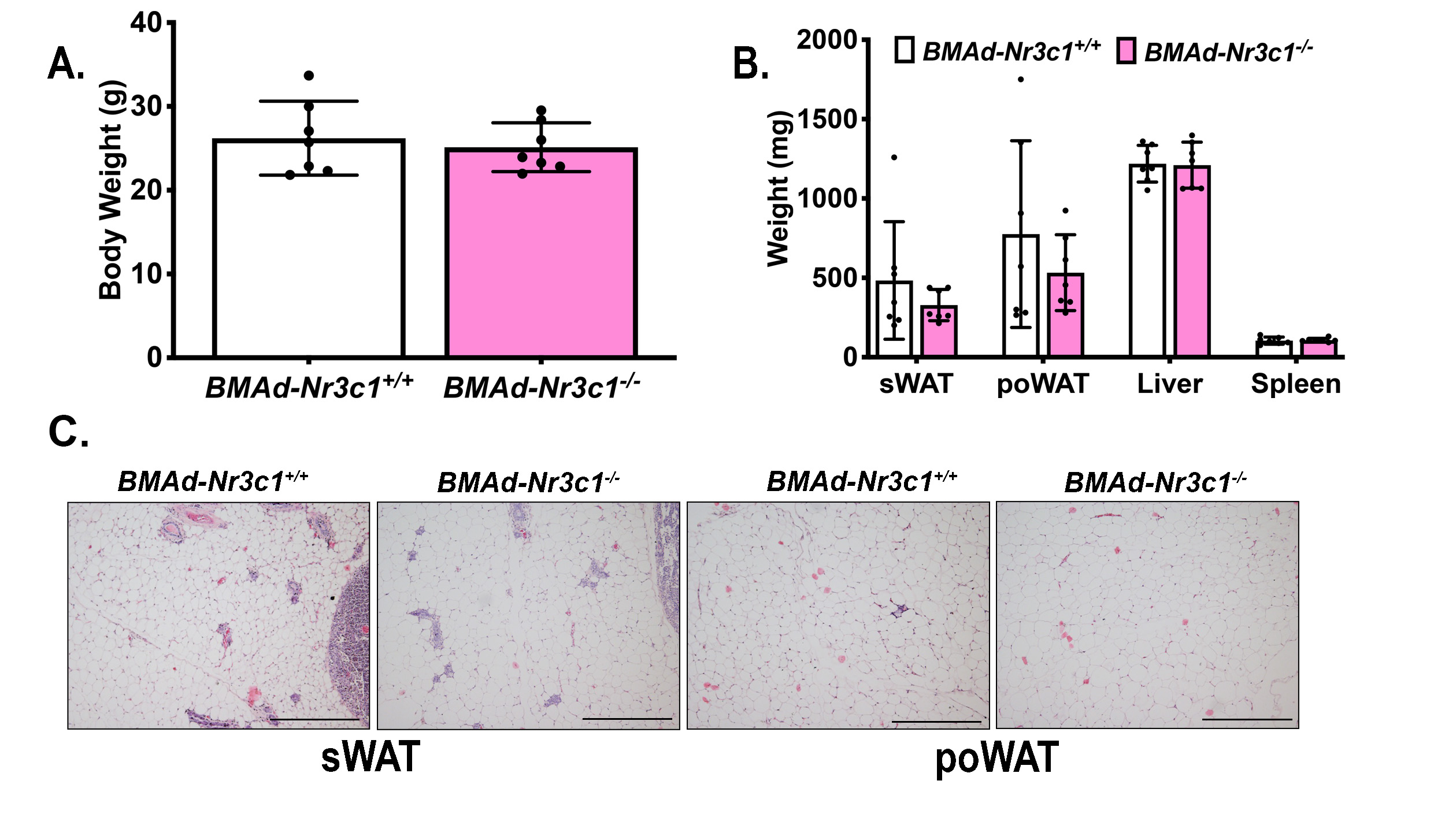

Supplement: Supplemental Figure 1 — Deletion of GR from BMAds of female mice does not alter WAT, liver, or spleen weights. Female mice at 31–39 weeks of age were euthanized and (A) body weights and (B) tissue weights of sWAT, poWAT, liver, and spleen were determined. Statistical analysis was performed using an unpaired t-test. (C) sWAT and poWAT were fixed, paraffin-sectioned, and stained with H&E. Scale bar: 0.22 mm. [file Image_1.jpeg]

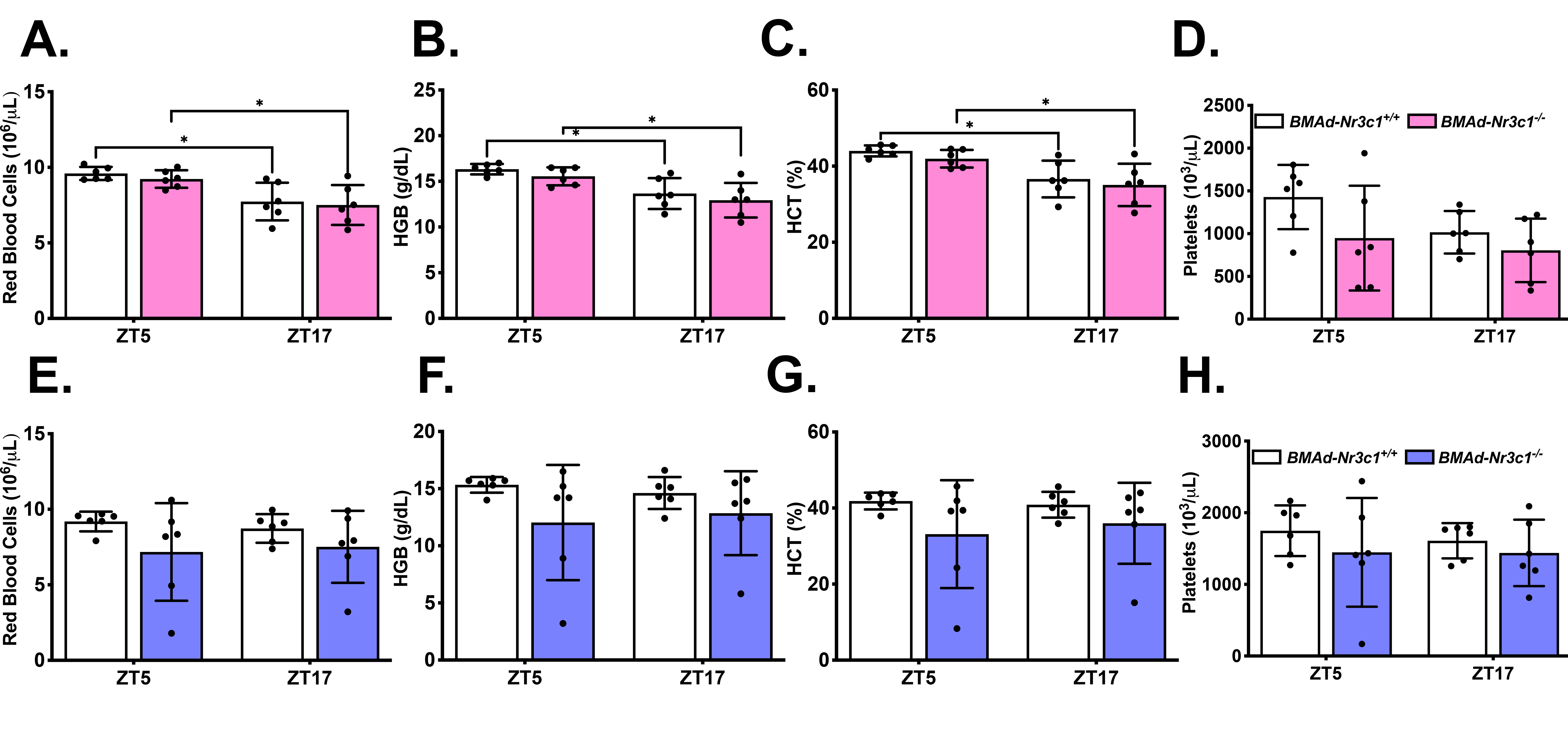

Supplement: Supplemental Figure 4 — Deletion of GR from BMAds does not alter circulating red blood cell populations. Circulating blood cell populations were measured in (A–D) female and (E–H) male BMAd-Nr3c1-/- and control mice at ZT5 and, following a 48-hour recovery period, at ZT17. Statistical analysis was performed using a one-way ANOVA. *p<0.05. [file Image_4.jpeg]
